# Effective Communication of Stroke Signs in Spanish to Patients in Cognitive Impairment

**DOI:** 10.1002/alz.093533

**Published:** 2025-01-09

**Authors:** Neeraj Singh

**Affiliations:** ^1^ Northwell Health, Great Neck, NY USA

## Abstract

**Background:**

Strokes affect 15 million people worldwide annually, including 5% of patients over the age of 75, making them a leading cause of death and disability, especially in elderly patients with cognitive impairment. Mnemonics have been developed in multiple languages to inform the public how to recognize stroke signs, including the English “BEFAST.” For Spanish‐speaking populations, the mnemonics “AHORA” and “RAPIDO” are both circulated with pictorial brochures. This study compares the effectiveness of both mnemonics in helping patients with cognitive impairment recognize stroke signs.

**Method:**

Between January and December 2023, 50 Spanish‐speaking patients with cognitive impairment or any type of dementia were interviewed in the Northwell Health Neurosciences clinic in New York. Each participant was shown the AHORA and RAPIDO brochures and asked to identify for each handout its purpose, favorable features, and ranking of quality (0‐5). To mitigate presentation bias, half of the participants viewed the AHORA brochure first, while the other half viewed the RAPIDO brochure first. A paired t‐test was conducted to determine the statistical significance of the difference in average ranking of each brochure.

**Result:**

The AHORA brochure received an average ranking of 3.66/5.00, significantly higher than the average ranking of 3.36/5.00 for the RAPIDO brochure (p = 0.0096). Positive comments about the AHORA brochure included the brochure appearing concise, the mnemonic being shorter, and the emphasis on calling 911 when stroke signs are recognized. Comments about the RAPIDO brochure included the brochure containing excessive information and the mnemonic being longer and more difficult to understand.

**Conclusion:**

Participants preferred the AHORA brochure over the RAPIDO brochure for its concise appearance and descriptions of each stroke sign, and emphasis on calling 911 when stroke signs are recognized. These findings provide valuable guidance for physicians in selecting brochures for Spanish‐speaking patients and patients with cognitive impairment, and offer insights into the elements necessary to develop effective mnemonics in other languages.

